# Transcriptomic Analysis Reveals Differential Expression of Genes between Lung Capillary and Post Capillary Venules in Abdominal Sepsis

**DOI:** 10.3390/ijms221910181

**Published:** 2021-09-22

**Authors:** Milladur Rahman, Zhiyi Ding, Carl-Fredrik Rönnow, Henrik Thorlacius

**Affiliations:** Department of Clinical Sciences, Malmö, Section for Surgery, Lund University, 214 28 Malmö, Sweden; milladur.rahman@med.lu.se (M.R.); zhiyi.ding.3635@med.lu.se (Z.D.); carl-fredrik.ronnow@med.lu.se (C.-F.R.)

**Keywords:** endothelial cells, lung, RNAseq, inflammation, sepsis

## Abstract

Lung endothelial cell dysfunction plays a central role in septic-induced lung injury. We hypothesized that endothelial cell subsets, capillary endothelial cells (capEC) and post capillary venules (PCV), might play different roles in regulating important pathophysiology in sepsis. In order to reveal global transcriptomic changes in endothelial cell subsets during sepsis, we induced sepsis in C57BL/6 mice by cecal ligation and puncture (CLP). We confirmed that CLP induced systemic and lung inflammation in our model. Endothelial cells (ECs) from lung capillary and PCV were isolated by cell sorting and transcriptomic changes were analyzed by bioinformatic tools. Our analysis revealed that lung capEC are transcriptionally different than PCV. Comparison of top differentially expressed genes (DEGs) of capEC and PCV revealed that capEC responses are different than PCV during sepsis. It was found that capEC are more enriched with genes related to regulation of coagulation, vascular permeability, wound healing and lipid metabolic processes after sepsis. In contrast, PCV are more enriched with genes related to chemotaxis, cell–cell adhesion by integrins, chemokine biosynthesis, regulation of actin filament process and neutrophil homeostasis after sepsis. In addition, we predicted some transcription factor targets that regulate a significant number of DEGs in sepsis. We proposed that targeting certain DEGs or transcriptional factors would be useful in protecting against sepsis-induced lung damage.

## 1. Introduction

Sepsis is a dominating cause of hospital-related mortality all over the world [1]. The management of sepsis patients is largely confined to supportive care which is partly due to an incomplete understanding of the pathophysiology [2]. The annual cost for sepsis is EUR 7.6 billion in Europe [2] and USD 24 billion in USA [3]. The substantial costs and lack of effective therapies in sepsis have urged a more thorough understanding of immune pathology of sepsis in order to develop therapies that will significantly modulate the outcome of the disease. Sepsis usually leads to single organ dysfunction, typically acute lung injury, followed by multiple organ dysfunctions [4]. Lung dysfunction is considered to be an insidious part of sepsis. Under resting conditions, endothelial cells (EC) play a pivotal part in maintaining normal homeostasis. However, during sepsis almost all of the aspects of EC function, including barrier function, vasoregulation and homeostasis, become dysregulated [5,6,7]. One pathophysiological hallmark of sepsis is excessive tissue infiltration of leukocytes and disruption of gaseous exchange [6,7]. We and others have previously shown that leukocyte recruitment into the lung tissue is a multistep process, involving tethering, rolling, firm adhesion and extravascular migration, mediated by specific adhesion molecules expressed on leukocytes and ECs [8,9]. For example, the selectin family of adhesion molecules, including P, E- and L-selectin, supports leukocyte tethering and rolling on vascular ECs in an organ-specific manner [10].

Another hallmark of sepsis is dysfunctional coagulation. It is well known that sepsis-induced mortality is greatly enhanced in the presence of disseminated intravascular coagulation [11]. During inflammation, the vascular endothelium becomes procoagulant and prothrombotic, causing excessive coagulation leading to microcirculatory tissue perfusion failure and organ damage [12,13]. Several therapies targeting EC function, such as recombinant human tissue factor pathway inhibitor, recombinant-activated protein C, recombinant-soluble thrombomodulin, protein C concentrate and heparin have been tested in previous trials, with no significant effect on clinical outcome [5,14]. A possible explanation to this could be that sepsis manifests itself into multiple processes, making the condition difficult to treat by targeting a single process. Additionally, concerns have been raised on the rapid dissemination of knowledge originating from lethal toxin-based animal studies to clinical trials without investigating the pathophysiological aspects further in experimental sepsis models. It is our expectation that a global analysis of adhesion molecules, cytokines and coagulation factors, as well as identification of transcriptional factors involved in dysregulation of EC, will provide unique insight in the chain reactions of sepsis, facilitating the next generation therapeutic regimens in sepsis.

The microvascular bed is composed of small arterioles, capillaries and postcapillary venules with a diameter less than 50 µm [15]. Early studies revealed that there are fundamental structural variations between microvascular beds in different organs primarily in microvascular patterns and capillary ultrastructure. For example, capillaries are fenestrated in the liver and kidney but not in the lung, heart and brain [16]. In the present study, we intended to investigate transcriptional changes and regulation in terms of inflammation, barrier function and coagulation in capEC and PCV of lung microvasculature, in an experimental model of sepsis. Our analysis revealed different transcriptomic changes in specific lung-derived EC and predicted some transcriptional factors that could be targeted in order to protect against sepsis-induced lung damage and dysfunction.

## 2. Results

### 2.1. Lung capEC and PCV Are Transcriptionally Different

**Figure 1:**
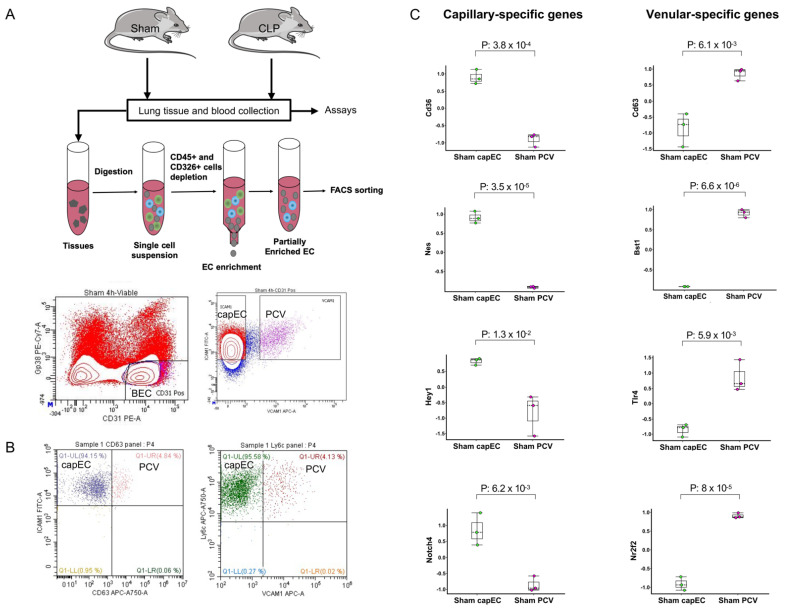
Lung endothelial subsets isolation and transcriptional signature genes. (**A**) Upper panel showing experimental design; middle panel showing lung tissue digestion and EC enrichment by depletion of CD45+ and CD326+ cells; lower panel showing flow cytometry gating strategy for the isolation of capEC and PCV from CD31+gp38− blood ECs (BECs) of lungs through the use of antibodies against Icam1 and Vcam1. (**B**) Identity of blood capEC and PCV was further confirmed by flow cytometry using Ly6c and venular-specific CD63 markers. (**C**) Expression of capillary-specific signature genes (*Cd36, Nes, Hey1* and *Notch4*) in CD31+Icam1+Vcam1- BECs and venular-specific signature genes (*Cd63, Vcam1, Tlr4* and *Nr2f2*) in CD31+Icam1+Vcam1+ BECs. Samples were collected 4 h after sham operation. Box plots represent the median (25–75 percentile) and the whiskers extend from the minimum to the maximum levels and dots represent sample values, normalized to mean = 0, variance = 1. *p*-value indicates statistical significance level.

For transcriptomic analysis of lung EC subsets, we sorted EC subsets using a modified protocol [17] described details in the methods section. We sorted blood capillary and post capillary venular ECs through the use of monoclonal antibodies. The capEC subset was identified as CD31+Icam1+Vcam1- and PCV subset was identified as CD31+Icam1+Vcam1+ (Figure 1A). The identity of capEC and PCV were further confirmed by two other antibody panels where capEC was identified as CD31+Icam1+CD63- and CD31+Ly6c+Vcam1-; PCV was identified as CD31+Icam1+CD63+ and CD31+Ly6c+Vcam1+ (Figure 1B).

Flow cytometry analysis revealed that CD63, a tetraspanin protein linked to leukocyte adhesion and recruitment [18], was low on capEC but high on PCV. Icam1 is known to be expressed to a high degree in capEC and to a low degree in arteries [19]. In peripheral lymph nodes and peyer’s patch, Vcam1 was found to be highly expressed in post capillary venules (known as HECs) but very low or undetectable in capEC [17]. By flow cytometric analysis, we found that lung BECs uniformly expressed Icam1 and Ly6c and use of Vcam1 and CD63 antibodies together with Icam1 or Ly6c antibodies separate lung capEC from PCV. capEC and PCV separation was better using Icam1 and Vcam1 markers than any other combinations, therefore, we sorted ECs (CD31+gp38-) with high expression of Icam1 and low expression for Vcam1 as capEC and ECs with high expression of Vcam1 and Icam1 as PCV. The purity of the sorted capEC was confirmed to be 97.5% (Supplementary Figure S1). Gene expression analysis of sham capEC and PCV revealed that ECs sorted as capEC expressed capillary-specific signature genes, including *CD36, Nes, Hey1* and *Notch4* (Figure 1C). On the other hand, BECs sorted as PCV expressed venule-specific signature genes, including *CD63, Bst1, Tlr4* and *Nr2f2* (Figure 1C), suggesting that sorted capEC and PCV are transcriptionally different as observed by Lee M, et al. [17]. Principal component analysis (PCA) showed that biological replicates of capEC and PCV cluster together (Supplementary Figure S2A), suggesting that samples in capEC have similar expression but different than PCV group. Top 100 DEGs of sham capEC and PCV were displayed in a PCA plot (Supplementary Figure S2B) (*p* ≤ 1.9 × 10^−15^, padj ≤ 2.9 × 10^−13^; > 4-fold difference). Top 100 DEGs in sham capEC and PCV in a PCA plot also suggested that lung capEC and PCV are transcriptionally different (Supplementary Figure S2C). For example, capEC were found to be enriched with genes involved in cell proliferation and angiogenesis, such as *Dpp4, Nes, Kitl* and *Kit* (Supplementary Figure S2C). In contrast, PCV were found to be enriched with genes involved in cell adhesion and migration, such as *Bst1, ptk2b, Vwf, Amigo2, Itgb3, Itgb4* and *Cdh13* (Supplementary Figure S2C). PCV were also found to be enriched with master venous regulator *Nr2f2* in the top 100 genes.

### 2.2. Sepsis Induces Pulmonary and Systemic Inflammation

**Figure 2:**
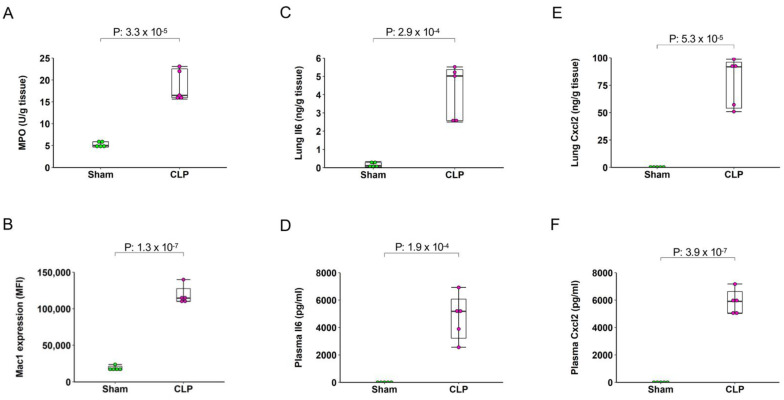
Tissue and systemic inflammation after induction of abdominal sepsis. (**A**) Pulmonary MPO; (**B**) Il6; (**C**) Cxcl2; (**D**) Mac1 expression on circulatory neutrophils; (**E**) plasma Il6 and (**F**) plasma Cxcl2. Samples were collected 4 h after sham and CLP. Box plots represent the median (25–75 percentile) and the whiskers extend from the minimum to the maximum levels and dots represent sample values and *n* = 5. *p*-value indicates statistical significance level.

To confirm pulmonary and systemic inflammation 4 h after CLP, we performed biochemical and immunological assays. MPO (an indicator of neutrophils) and Mac1 expression on the surface of circulating neutrophils were examined by enzymatic assay and flow cytometry, respectively. We observed that pulmonary MPO activity increased by more than 3-fold in CLP animals (Figure 2A). Flow cytometry analysis revealed that Mac1 expression on circulating neutrophils increased by more than 6-fold in CLP animals (Figure 2B). Moreover, CLP markedly increased pulmonary and plasma formation of Cxcl2 and Il6 (Figure 2C–F), suggesting that 4 h CLP indeed caused clear-cut pulmonary and systemic inflammation.

### 2.3. Differential Gene Expression between capEC and PCV during Sepsis

**Figure 3:**
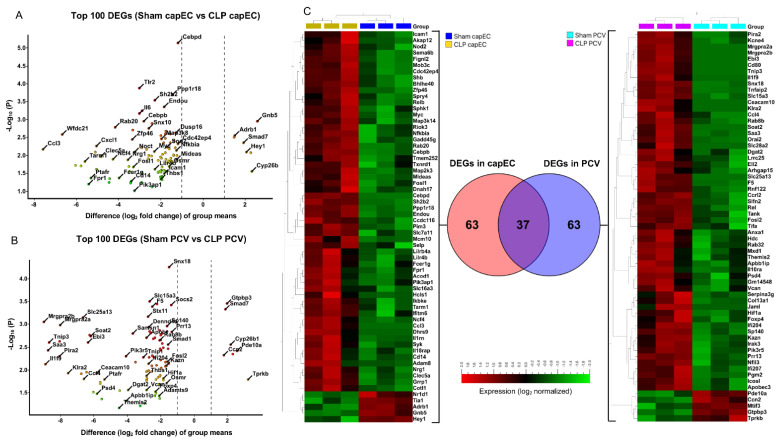
Top 100 differentially expressed genes (DEGs) in lung EC subsets. (**A**) Volcano plot showing top 100 DEGs in the capEC (sham capEC vs. CLP capEC), *p* ≤ 3.4 × 10^−6^, padj ≤ 0.0004. (**B**) Volcano plot showing top 100 DEGs in the PCV (Sham PCV vs. CLP PCV), *p* ≤ 1.9 × 10^−6^, q ≤ 0.0002. Volcano plot is constructed by plotting the negative log10 of the *p* value versus the group mean difference (log2 fold change). Variables are colored based on fold change, blue dots indicate upregulated in CLP and red dots indicate downregulated n CLP. Highly differed variables are in the top left and top right areas of the volcano plot. (**C**) Venn diagram showing overlapping of top 100 DEGs between capEC and PCV (middle panel), 84 different variables in capEC are shown in left heatmap and 84 different variables in PCV are shown in right heat map. Hierarchical clustering and box color of heat map are based on data normalization, mean = 0, variance = 1. *p*-value indicates statistical significance level and q-value indicates false discovery rate.

Our analysis revealed that, lung EC subsets had differential expression of genes involved in different types of biological functions. In the top 100 DEGs of capEC (sham vs. CLP), we observed expression of genes related to cellular response to lipopolysaccharide (*Cebpb, Cd14, Litaf, Adam9, Acod1, Il6, Icam19*), protein phosphorylation (*Nuak2, Riok3, Smad1, Ccl3, Map2k3, Pim3, Syk*), apoptotic process (*Litaf, Nuak2, Birc3, Tia1, Shb, Tnfaip3*), blood vessel morphogenesis (*Shb, Thbs1, Syk*) and positive regulation of cell extravasation (*Adam8, Icam1, Ptafr*) (Figure 3A and Supplementary Material S1). In contrast, top 100 DEGs of PCV (sham vs. CLP), expressed genes linked to cellular response to lipopolysaccharide (*Wfdc21, Cxcl1, Il10ra, Irak3, Noct, Sod2, Trib1, Litaf*), acute-phase response (*Il6, Serpina3n, Saa3*), positive regulation of tumor necrosis factor production (*Ccl4, Ptafr, Tlr2*), cytokine-mediated cell signaling (*Il1fa, Il6, Irak3, Socs2*), response to hypoxia (*Fosl2, Hif1a, Sod2, Tlr2*) and neutrophil chemotaxis (*Amica1, ccl4, Cxcl1*) (Figure 3B and Supplementary Material S2). We compared the top 100 DEGs between capEC (from sham vs. CLP) and PCV (from sham vs. CLP) and found that gene expression between them was different. It was observed that in the top 100 DEGs, capEC and PCV had 37 genes in common (*Adam9, Adamts9, Apobr, Bcl3, Birc3, Cxcl1, Cyp26b1, D10Wsu102e, Dennd4a, Dusp16, Gramd1a, Il6, Litaf, Map3k8, Noct, Nuak2, Osmr, Pfkfb3, Ptafr, Samsn1, Sema4c, Serpina3f,, Serpina3g, Smad1, Smad7, Snx10, Socs2, Sod2, Stx11, Susd6, Thbs1, Tlr2, Tnfaip3, Tnip1, Trib1, Wfdc21, Zfp46*) mainly involved in immune response and apoptosis, whilst 84 genes differed (capEC left panel, PCV right panel, Figure 3C). PCA analysis showed that biological replicates of sham capEC cluster together, same for biological replicates of CLP capEC, sham PCV and CLP PCV (Supplementary Figure S3). The first principal component (largest difference) separates Sham PCV from CLP PCV, indicating that CLP caused massive transcriptional changes in PCV (Supplementary Figure S3E). Second principal component separates capEC from PCV, indicating endothelial subsets specific transcriptional differences. Third principal component (lowest difference) separates sham capEC from CLP capEC, indicating CLP caused relatively less transcriptional changes in capEC (Supplementary Figure S3E). We also compared the top 500 genes and found only 185 common genes between capEC and PCV DEGs (not shown), confirming that CLP caused distinct transcriptional changes in capEC and PCV.

### 2.4. Distinct Gene Sets and Pathways Enrichment between capEC and PCV

**Figure 4:**
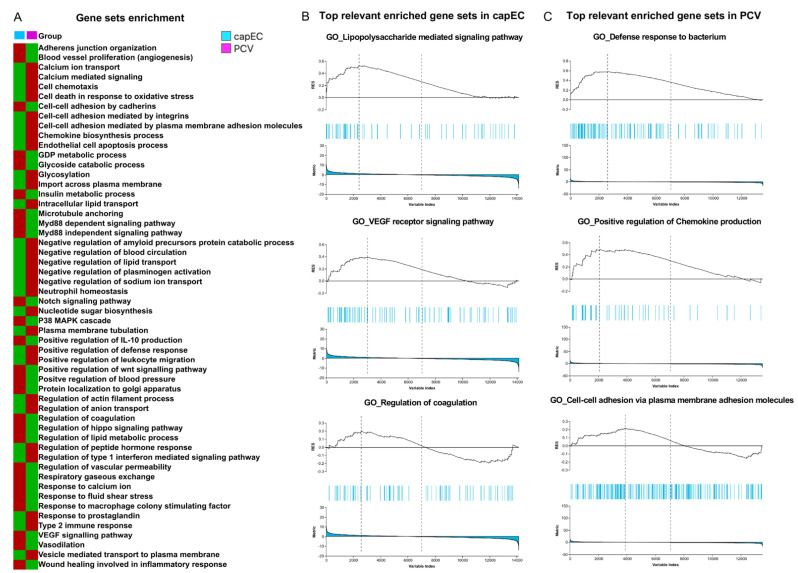
Gene sets enrichment analysis. (**A**) Heat map showing differentially enriched GO pathways based on normalized enrichment score in lung capEC and PCV after induction of sepsis, red indicates higher gene sets enrichment and green indicates lower gene sets enrichment. (**B**) Top three relevant enriched gene sets (human orthologs) in lung capEC. (**C**) Top three relevant enriched gene sets (human orthologs) in lung PCV. For GSEA analysis, mouse genes are converted to human orthologs as described in the materials and methods section. GSEA analysis was performed using gene sets derived from the GO Biological Process (BP) ontology of the Molecular Signatures Database (v7.2 MSigDB). Plots show running enrichment score (RES), the match positions for the genes (blue lines) in the selected data set and the ranking metric values for the input gene list.

**Figure 5:**
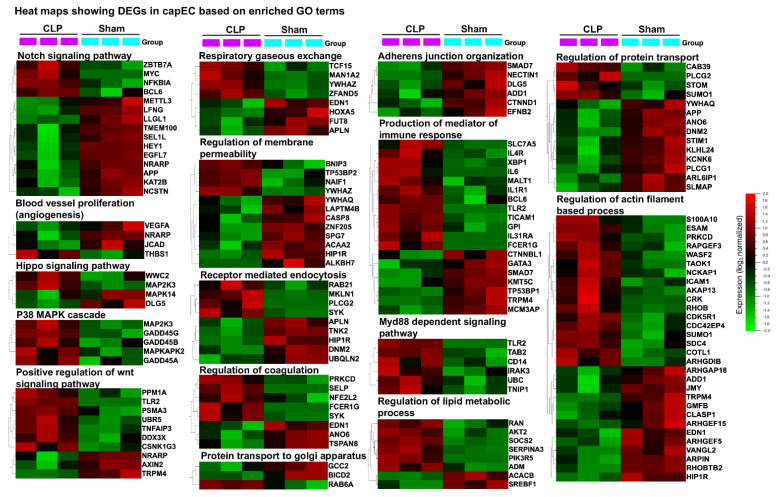
Heat maps showing DEGs (*p* ≤ 0.05) with GO enriched term in capEC. Samples were collected 4 h after induction of abdominal sepsis. GO terms were based on biological process of Molecular Signatures Database (v7.2 MSigDB). For GSEA analysis, mouse genes were converted to human orthologs as described in the materials and methods section. Hierarchical clustering of variables and box color of heat maps were based on data normalization, mean = 0, variance = 1.

**Figure 6:**
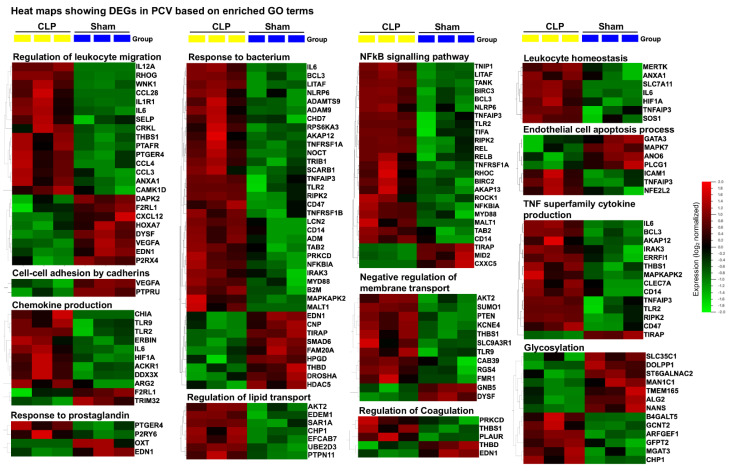
Heat maps showing DEGs (*p* ≤ 0.05) with GO enriched term in PCV. Samples were collected 4 h after induction of abdominal sepsis. GO terms were based on biological process of Molecular Signatures Database (v7.2 MSigDB). For KEGG pathway analysis, mouse genes were converted to human orthologs as described in the materials and methods section. Hierarchical clustering of variables and box color of heat map was based on data normalization, mean = 0, variance = 1.

Next, we performed Gene Set Enrichment Analysis (GSEA) using predefined sets of genes based on functional annotation of DEGs. Our analysis revealed discordance between capEC (sham vs. CLP) and PCV (sham vs. CLP). We selected and compared the most significantly enriched signaling pathway, based on the Normalized Enrichment Score (NES) for capEC and PCV, respectively (Figure 4A). Our analysis revealed that sepsis caused higher gene sets enrichment in capEC compared with PCV in the following GO terms: adherens junction organization, angiogenesis, GDP metabolic process, glycoside catabolic process, insulin metabolic process, lipid metabolic process, notch signaling pathway, Wnt signaling pathway, VEGF signaling, plasma membrane tubulation, regulation of coagulation, regulation of vascular permeability and wound healing involved in inflammatory response (Figure 4A). Concurrently, sepsis caused higher gene sets enrichment in PCV in the following GO terms: cell chemotaxis, cell–cell adhesion by cadherins, cell–cell adhesion by integrins, chemokine biosynthesis process, calcium-mediated signaling, negative regulation of blood circulation, negative regulation of plasminogen activation, negative regulation of sodium ion transport, regulation of actin filament process, neutrophil homeostasis and response to prostaglandin (Figure 4A). Three selected gene sets from capEC (Figure 4B) and three from PCV (Figure 4C) are presented to show running enrichment score (RES). Selected DEGs of capEC based on relevant GO terms (Figure 5) and selected DEGs of PCV based on relevant GO terms (Figure 6), were presented as heatmaps.

KEGG pathway enrichment analyses using the top 500 DEGs revealed that both capEC and PCV have similar types of pathway enrichment (Table 1 and Table 2). In the DEGs of capEC, the most enriched pathway was TNF signaling pathway (Top upregulated TNF signaling-related genes were shown in Supplementary Figure S4), followed by NOD-like receptor signaling pathway, Toll-like receptor signaling pathway, apoptosis pathway, NF-kappa B signaling pathway, cytokine-cytokine receptor interaction, MAPK signaling pathway, p53 signaling pathway, TGF-beta signaling pathway and chemokine signaling pathway. In the DEGs of PCV, the most enriched pathway was TNF signaling pathway, followed by NF-kappa B signaling pathway (top upregulated NF-kappa B signaling related genes are shown in Supplementary Figure S5), Toll-like receptor signaling pathway, NOD-like receptor signaling pathway, cytokine-cytokine receptor interaction, apoptosis, cell adhesion molecules, JAK-STAT signaling pathway, HIF-1 signaling pathway and TGF-beta signaling pathway.

Our analysis also revealed that several transcription factors, such as, *Rel* (also known as *cRel*), *Relb, Cebpb, Cebpd, Nfkbia, Nfkbie* and *Myc*, were significantly upregulated in both capEC and PCV (Supplementary Materials S1 and S2). By use of Qlucore software 3.6 (33) (Qlucore AB, Lund, Sweden) we also predicted some transcription factor targets (TFTs) those found to regulate many DEGs (*p* ≤ 0.05, difference of at least 2-fold) both in capEC and PCV. Our findings suggest that transcription factor such as Crel, NF-kB65, Znf791, Cebpb and miR-649 might regulate most DEGs of capEC during sepsis (Table 3). For example, Crel was found to regulate genes involved in inflammation: *Rel, Relb, Birc3, Bcl3*; regulation of transcription: *Nfkbia, Rel, Relb, Stat6, Tcf7l2*; response to cytokine: *Rel, Relb*; regulation of focal assembly: *S100a10, Sdc4*; regulation of apoptosis: *Nfkbia, Birc3, Ier3, Map3k8, Ube2d3*. In PCV, we found that transcription factors such as Cebpb, Gata6 and several miRs such as miR-4769-3p, miR-362-5p, miR-510, might regulate most DEGs of PCV during sepsis (Table 2). For example, Gata6 was found to regulate genes involved in transcription process: *Bcl6, Ctcf, Gpbp1, Lhx6, Smad5, Fosl2, Sall2*; nodal signaling pathway: *Tgif1, Tgif2*; Atypical chemokine receptor: *Ackr1* that acts as chemokine receptor for Ccl2, *Ccl5, Ccl7, Ccl11, Cxcl5, Cxcl6, Il8* (human)/*Cxcl2* (mouse); regulation of ubiquitination: *Mid1* (E3 ubiquitin-protein ligase midline-1) that regulates innate immune response via NF-kB signaling.

### 2.5. Validation by qRT-PCR

**Figure 7:**
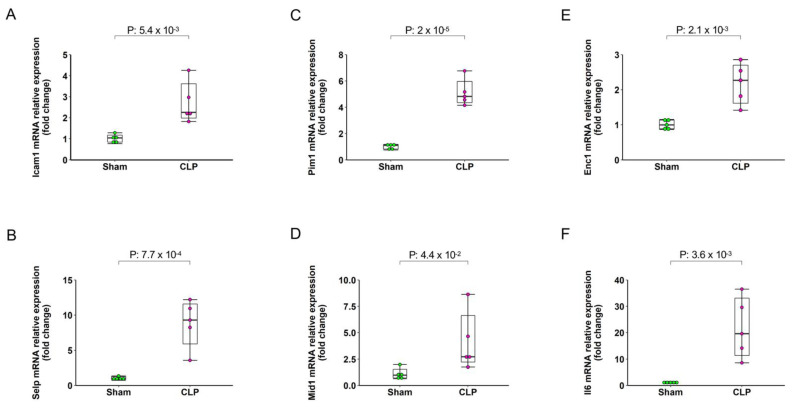
Validation of selected RNA-seq data by qRT-PCR. Samples were collected 4 h after induction of abdominal sepsis. Relative expressions of (**A**) *Icam1*, (**B**) *Selp*, (**C**) *Pim1*, (**D**) *Mid1*, (**E**) *Enc1* and (**F**) *Il6* mRNAs were quantified using qRT-PCR where GAPDH was used as a housekeeping gene for mRNA and expressions were determined using 2^−ΔΔCT^ method. Box plots represent the median (25–75 percentile) and the whiskers extend from the minimum to the maximum levels and dots represent sample values and *n* = 5.

To identify possible technical artifacts in our RNAseq data and to investigate whether the tissue processing for single-cell preparation have any impact on gene expression, we performed qRT-PCR of six randomly selected genes (*Icam1, Pim1, Enc1, Selp, Mid1* and *Il6*) in freshly collected lung samples. The qPCR results showed that all selected genes consistently changed in the lung samples of CLP animals (Figure 7).

## 3. Discussion

Despite significant investigational efforts, sepsis remains a major health-care concern with a high mortality rate in intensive care units [20]. The treatment of sepsis is largely supportive apart from antibiotic therapies. Several promising anti-inflammatory and anti-coagulant drugs have failed to improve septic survival in clinical trials [5,14,21]. The reason could be that sepsis turns itself into a multiple process making the therapeutic process difficult to treat with a single target. Hence, in the present study, we investigated global expressional changes in lung EC subsets during sepsis for the first time. We identified several groups of DEGs and pathways in capEC and PCV in sepsis, including genes involved in innate immune response, regulation of vascular permeability, regulation of coagulation, respiratory gaseous exchange, EC apoptosis, cell chemotaxis, cellular junction organization, protein transport and relevant signaling pathways. We also identified several transcription factors and miRNAs which could be targeted to reduce global inflammatory and metabolic changes in sepsis.

Our analysis revealed that sorted sham lung capEC and PCV expressed distinct signature gene sets. We observed that lung capEC are highly enriched with *Cd36, Hey1, Notch4* and progenitor marker, such as, *Nes*, while in PCV, we observed high expression of *CD63, Bst1, Tlr4* and *Nr2f2* genes. These observations are in line with two other studies showing that in lymphoid tissue capEC and venous high EC express distinct signature genes [17,19]. We found that capEC are enriched with genes involved in specialized functions, such as, vasculogenesis: *Acvrl1, Cd34*; artery morphogenesis: *Hey1, NPRL3*; plasma membrane transport: *Rab31, Rsc1a1*; and regulation of blood circulation: *Apln, Casr, Fga*. In contrast, PCV was enriched with genes involved in leukocyte trafficking: *Bst1, Vcam1, Jam1*; and cytokine secretion process: *Il33, Il6, Lbp*. However, both capEC and PCV were highly enriched with genes related to coagulation: *Cd36, Fga, Fibp, Prkce*; and *Anxa5, Gata6, Prkg1, Vwf, Sh2B2*, respectively. Based on the differences we observed in sham capEC and PCV, we anticipated that the responses would be different during sepsis.

Lung microvessels are not only involved in transportation of nutrients and oxygen into tissues, but also actively involved in interactions with circulating cells (i.e., leukocytes, platelets, etc.), as well as secretion of mediators into the circulation and local tissues [14]. Our analysis revealed that sepsis caused upregulation or downregulation of the set of genes involved in regulation of membrane permeability, gaseous exchange, cellular junction, cytokine production, leukocyte migration, cell apoptosis, actin polymerization and protein transport across plasma membrane. A hallmark of sepsis is exudation of fluid and proteins from the intravascular space into the extravascular space of the lung. Experimental sepsis studies suggest that loss of fluid from blood vessels is caused by the breakdown of EC barrier function [22,23] and/or direct EC injury [24]. In the present study, we observed significant upregulation of genes, such as, *Bnip3, naif1, Tb53bp1* and *Ywhaz*, and downregulation of genes, such as *Casp8, Spg7, Acaa2, Hip1r* and *Ywhaq*, in capEC related to vascular permeability. Notably, Bnip3 (Bcl2 interacting protein 3) has been shown to regulate human microvascular EC death [25] and overexpression of Naif1 (Nuclear apoptosis-inducing factor 1) has been reported to increase apoptosis of cancer cells [26]. Taken together, these studies support our finding that both Bnip3 and Naif1 are involved in EC death and permeability in sepsis. From the downregulated gene sets, we found downregulation of *Acaa2* (Acetyl-CoA acyltransferase 2), which is known to inhibit mammary epithelial cell apoptosis [27], thus downregulation of *Acaa2* during sepsis may increase apoptosis of capEC. EC permeability was related to other genes, such as, *Cldn5* (claudin 5) and *Pecam-1*/CD31 (platelet EC adhesion molecule), also significantly upregulated in septic capEC, suggesting that sepsis induces gene expression related to capillary permeability. Although the function of other differentially expressed genes are not investigated in sepsis yet, it could be hypothesized that they all act in concurrence to damage microvessels during sepsis. Increase in protein and water transport during sepsis could also occur due to increase in transcellular transport and disruption of adherens junction [28]. In fact, in our data, we have observed downregulation of genes related to both adherens junction organization (*Smad7, Nectin7, Dlg5, Add1, ctnnd1, Efnb2*) and genes related to regulation of protein transport (*App, Dmn2, Ano6, Kcnk6, Slmap*). In addition, a large number of genes related to actin polymerization were changed during sepsis in the present study. This notion is supported by the fact that actin polymerization is a key regulator of EC gap formation and permeability during inflammation [29]. We also observed differential expression of genes related to Notch, Wnt and Hippo signaling. These observations are also in line with other studies showing that all these signaling pathways are involved in sepsis-induced lung inflammation [30,31,32]. Similar to capEC of lymph nodes [19], lung capEC in this study is also found to be featured with proangiogenic genes. Hence, sepsis caused downregulation of proangiogenic genes such as *Vegfa, Nrarp, Jcad* and upregulation of the anti-angiogenic gene *Thbs1*, indicating that the proliferative function of capEC might be disrupted in sepsis.

Moreover, circulatory monocytes and endothelial tissue play a key role in regulating blood coagulation and hemostasis [33,34,35]. We found that CLP caused upregulation of genes, such as *Prkcd, Nfe2l2, Fcer1g* and *Syk* and downregulation of genes, such as *Edn1, Ano6* and *Tepan8*, which are closely linked to the coagulation cascade. Most of these genes, involved in coagulation, have not been examined in models of sepsis previously. Furthermore, plasminogen activator inhibitor-1 (Pai-1) is known to regulate fibrinolysis by inhibiting tissue plasminogen activator (tPA) and an increase in Pai-1 is associated with fibrin deposition in the lung [36]. In our data, we observed an increase in *Serpine1* (gene responsible for Pai-1 production), suggesting that profibrinolytic properties of capEC is also disrupted and coagulation cascade might be activated, during sepsis. Although gene enrichment analysis revealed that response of capEC in terms of coagulation is higher than PCV, PCV also expressed a significant number of coagulation related genes. For example, we observed a significant increase in genes encoding *F2rl2, F5, Thbs1, Serpind1* in lung PCV, suggesting that both PCV and capEC are important for coagulation cascade activation in sepsis.

Moreover, activation and dysfunction of microvascular EC occur as a result of exposure to pathogens and inflammatory mediators, as well as by adherent leukocytes and platelets [37,38]. In response to pathogens or damage associated molecular patterns (DMAPs), EC secrete cytokines and chemokines [39]. We found upregulation of *Cxcl2* (macrophage inflammatory protein 2, Mip-2), *Tnfrsf1b* (Tnf Receptor Superfamily Member 1B), *Ccl3* (Mip-1-alpha), *Ccl4* (Mip-1β), *Il6* (interleukin 6) and *Il12* (interleukin 12) in septic PCV. In addition, receptor for Il1, *IL1r* was also highly upregulated in septic PCV in our study. Furthermore, we observed that *Cxc12* (stromal cell-derived factor 1) was downregulated in septic capEC.

Accumulating studies have shown that expression of cell adhesion molecules on EC and subsequent adhesion and migration of leukocytes via EC play a central role in septic-induced tissue injury [40,41,42]. Importantly, several experimental sepsis studies demonstrated that neutrophil infiltration through postcapillary venules is a rate limiting step [42,43,44]. In our study we observed significant upregulation of genes on the lung EC related to leukocyte migration. For example, genes related to leukocyte adhesion to EC, such as *Selp* (P-selectin), *Icam1* (intercellular adhesion molecule 1), were significantly upregulated in PCV during sepsis. In addition, we observed upregulation of *Sele* (E-selectin) in our data (not in the top 500). Activation of these selectins on endothelium has previously been shown to facilitate leukocyte and platelets binding via their surface glycoproteins [45,46]. We also observed significant enrichment of glycosylation pathway in PCV which is known to regulate leukocyte recruitment and inflammation [47]. Moreover, similar to other studies [48,49], we found a significant upregulation of Icam1 in capEC, suggesting that activated leukocytes might bind with Icam1 and plug into capEC and cause EC damage.

We identified several transcription factor targets of our DEGs, of which NF-κB transcription factor plays a pivotal role in modulation of immune response during sepsis [50]. Accumulating data suggest that homo- and heterodimers of NF-κB transcription factors, such as, p65, RelA, RelB, cRel, p50, p52, p65, play a major role in regulating genes involved in aggravated inflammatory responses, including synthesis of cytokines, coagulation factors and cell adhesion molecules [51,52]. An oligonucleotide decoy of transcription factor, NF-κB, has been shown to reduce acute lung injury in CLP-induced mouse model of sepsis [53]. Targeting transcription factors using small molecules is known to be difficult because of complicated protein–protein and/or protein-DNA interactions. Interestingly, recent progress in peptide-based drug targets has been shown to accomplish such challenges to some extent [54,55]. However, it should be noted that targeting a transcription factor that regulates many biological processes may simultaneously disrupt other critical physiological responses, and further research is needed before clinical use.

The spectrum of EC dysfunction during sepsis is so complex that a detailed presentation of all aspects of changes is not possible in this paper. Nevertheless, we tried to discuss important molecular changes covering, EC barrier dysfunction, activation of coagulation system in relation to EC, leukocyte-EC interactions in relation to lung failure. In addition to confirming genes previously identified in sepsis, we identified several novel genes and candidate transcription factors of potential importance in sepsis. The functional role of previously unaddressed genes as well as the possibility of using some of these genes as early markers of lung damage and targeting of candidate transcription factors should be subjects of future experimental and clinical studies.

## 4. Materials and Methods

### 4.1. Animal Experiments

All experiments were conducted in accordance with the legislation on the protection of animals and approved by the Regional Ethical Committee for Animal Experimentation at Lund University, Lund, Sweden (Permit number: 5.8.18-08769/2019). Male C57BL/6 mice (20–25 g) were obtained from Zanvier labs, Le Genest-Saint-Isle, France, and kept in a pathogen-free facility on a 12–12 h light-dark cycle with free access to food and tap water. Mice were housed at least one week to acclimate to the new surroundings. Animals were anesthetized with 75 mg of ketamine hydrochloride (Hoffman-La Roche, Basel, Switzerland) and 25 mg of xylazine (Janssen Pharmaceutical, Beerse, Belgium) per kg body weight. The ARRIVE guidelines were followed for all animal experiments [56]. Experimental lethal grade sepsis was induced by cecal ligation and puncture (CLP) as described elsewhere [57], and this model of sepsis is known to cause massive infiltration of neutrophils and lung injury [58,59]. Briefly, cecum was exposed, and feces were pushed towards the ascending colon. A ligature was made below the ileocecal valve covering 70% of the cecum, thereafter the cecum was punctured twice with a 21-gauge needle. Mice were returned to their cages and reanesthetized for sample collection 4 h after CLP procedure. For RNA-sequencing fresh samples were digested for single-cell suspension and lung EC were sorted out for total RNA extraction as described below. In separate experiment, blood and lung tissues were collected for biochemical and immunoassays as described below.

### 4.2. Endothelial Subsets Sorting and Total RNA Extraction

For EC subsets isolation, lung tissues were taken from the mice and digested to single-cell suspension. Briefly, tissues were minced in ice-cold HBSS and washed by centrifugation. Tissue pellets were then digested in HBSS media containing 0.2 mg/mL collagenase P, 0.8 mg/mL dispase II, 0.01 mg/mL DNase for 60 min at 37 °C with gentle rocking. Dissociated cell suspensions were collected in FBS to stop digestion then passed through 100 μm filter followed by 40 μm filter. ECs were enriched from the resulting cell suspensions by depletion of hematolymphoid cells and epithelial cells using anti-CD45 and anti-CD326 microbeads (Miltenyi Biotech, Bergisch Gladbach, Germany). Enriched ECs were labelled with antibodies for FACS sorting. Briefly, anti-CD31 was used as the primary defining EC marker and anti-gp38 to distinguish between lymphatic (gp38+CD31+) and blood (gp38-CD31+) EC. A pool of lineage markers (anti-CD45, -CD11a, -TER119, -EpCAM) and 7-AAD were used to exclude hematolymphoid, epithelial, stromal cells and dead cells from the analysis. CapEC subset was identified as CD31+Icam1+Vcam1- and PCV subset was identified as CD31+Icam1+Vcam1+. EC subsets were sorted directly into RLT buffer (Qiagen, West Sussex, UK). Total RNA was isolated from the sorted cells with RNeasy Plus Micro kit (Qiagen) and the quality of RNA was checked by Bioanalyzer RNA 6000 pico assay (Agilent, Santa Clara, CA, USA). Cell isolation, purification and sorting were conducted in the presence of RNase inhibitor (Sigma-Aldrich, Stockholm, Sweden) throughout the protocol.

### 4.3. RNA Sequencing and Data Analysis

For whole-transcriptome analysis, sequencing was performed at the Center for Translational Genomics (CTG), Lund University and Clinical Genomics Lund, SciLifeLab. Briefly, sequence library was prepared using SMARTer^®^ Stranded Total RNA-Seq Kit v2-Pico Input Mammalian (634411, Takara Bio USA Inc., Mountain View, CA, USA. After library preparation and quality check, the double-stranded-cDNA was sequenced on NextSeq 500 (SY-415-1001, Illumina) using (read one-index reads-read two, bp): 75-8-8-75 setup. The sequencing data was analyzed using the following pipeline: ‘Demultiplexing’ using the bcl2fastq2 software, ‘Raw Quality Control’ (QC) using FastQC, ‘Read Mapping and QC’ was performed using HISAT2 software, and the reference genome sequence was from the Ensemble database, the Mouse 38, and the annotation (GTF) was from the release 93, ‘Expression Counts’ was performed using the StringTie software and finally ‘Differentially Expressed Genes (DEGs)’ analysis was performed using DESeq2 in the statistical environment R (version 4.0.2). Variables with three or more samples having zero counts were filtered out before analysis. Volcano plots and heatmaps using DEGs were generated using Qlucore Omics Explorer software version 3.6 (33) (Qlucore AB, Lund, Sweden). Data were normalized to 2-logarithm and threshold was set to +1. Hierarchical clustering of variables and box color of heat maps was generated after data normalization to mean = 0, variance = 1.

### 4.4. Flow Cytometry

In a separate experiment, identity of capEC and PCV in enriched EC were confirmed by using additional capillary and venular markers. In one panel, anti-Icam1 and anti-CD63 antibodies were used to separate capEC and PCV from CD31+gp38- blood ECs (BECs) and in another panel anti-Ly6c and anti-Vcam1 antibodies were used to separate CD31+gp38- BECs. For Mac1 expression on blood neutrophils, blood was collected from the inferior vena cava in acid citrate dextrose (1:10) at 4 h after CLP induction. Cells were incubated with an anti-CD16/CD32 for 10 min to block FcγIII/IIRs and reduce non-specific binding. Then, cells were incubated with a phycoerythrin-conjugated anti-Ly6g (clone 1A8, BD Pharmingen, San Jose, CA, USA) antibody and an allophycocyanin (APC)-conjugated anti-Mac-1 (clone M1/70, integrin αM china, rat IgG2b, eBioscience Inc., San Diego, CA, USA) antibody at 4 °C for 20 min. Red blood cells were lysed and neutrophils were recovered following centrifugation. Flow cytometric analysis was conducted using Cytoflex flow cytometer (Becton Dickinson, Mountain View, CA, USA). Mac-1 expression (MFI, mean fluorescence intensity) was determined on gated Ly6g positive cells.

### 4.5. Myeloperoxidase (MPO) Assay

Polymorphonuclear leukocytes (PMNLs) are known to contain higher amounts of MPO than mononuclear leukocytes (MNLs) and frequently used as an indicator of PMNLs accumulation in the tissue [60,61]. For MPO assay, lungs were perfused with 10 mL PBS first and several pieces of lung tissue were snap frozen and stored at −80 °C for later assay. Frozen lung tissues were thawed and weighed and then homogenized in 1 mL mixture (4:1) of PBS and aprotinin (10,000 kallikrein inactivator units per ml; Trasylo, Bayer HealthCare AG, Leverkusen, Germany) for one minute. Homogenates were centrifuged for 10 min (15,300× *g*, 4 °C), and supernatants were frozen to −20 °C, and stored for other analysis. MPO activity was determined in the remaining pellets in accordance with an established protocol [60]. Pellets were resuspended in 0.2 M PB pH7.4, then centrifuged, the pellets were again suspended in one ml of 0.5% hexadecyl-trimethylammonium bromide buffer. The dissolved samples were frozen for 24 h, thawed, sonicated for 90 sec and put in a water bath (60 °C, 2 h). Enzyme reaction with substrate (3,3′,5,5′-Tetramethylbenzidine, TMB) (Sigma Aldrich, Stockholm, Sweden) as measured spectrophotometrically (450 nm, with a reference filter 540 nm, 25 °C). Data were expressed as MPO units per gram tissue.

### 4.6. ELISA

Plasma and lung levels of Il6 and Cxcl2 were quantified using double-antibody enzyme-linked immunosorbent assay kits (R&D Systems Europe, Ltd., Abingdon, Oxon, UK). For plasma, blood was collected from the inferior vena cava in acid citrate dextrose solution, and then centrifuged (2000× *g* for 10 min at 4 °C). After centrifugation, plasma samples were collected and stored at −20 °C for ELISA. For measurement of Il6 and Cxcl2 in lung tissue, lungs were perfused with 10 mL PBS first and several pieces of lung tissues were collected and snap frozen for later assay.

### 4.7. Gene Sets Enrichment Analysis and KEGG Pathway Analysis

In order to reveal and visualize biological processes involved in the pathogenesis of abdominal sepsis, we used Qlucore Omics Explorer software version 3.6 (33) (Qlucore AB, Lund, Sweden). For GSEA analysis, mouse gene symbols (first letter in upper case) were converted to human orthologs (all letters in upper case) using Ensembl databases by BioMart databases [62] in Bioconductor (version 3.12). GSEA analysis was performed using human orthologs which have corresponding mouse gene symbols. C5 gene ontology collection derived from the Molecular Signatures Database (MSigDB v7.2, Broad Institute, Cambridge, MA, USA) [63] was used for GSEA. Analysis was performed with a ranking metric based on two group comparisons. Qlucore GSEA displays Running Enrichment Score (RES), the match positions for the genes in the selected data set and the ranking metric values for the input gene list.

Kyoto Encyclopedia of Genes and Genomes (KEGG) pathway enrichment analyses were performed using the top 500 DEGs from capEC and PCV in Database for Annotation, Visualization and Integration Discovery (DAVID version 6.7; http://david.abcc.ncifcrf.gov) (accessed date: 26 March 2021) [64,65]. GO biological process terms were based on biological process of Molecular Signatures Database (v7.2 MSigDB). Mouse gene symbols were converted to human orthologs using Ensembl databases as described above and then used in DAVID. Hierarchical clustering of variables and box color of heat maps were generated after data normalization to mean = 0, variance = 1 in Qlucore program.

### 4.8. Validation by qRT-PCR

For the validation of RNA sequencing data, total RNA was extracted from freshly collected lung tissue by using TRIzol (Invitrogen, Thermo Fisher Scientific, Inc., Waltham, MA, USA) and purified using Direct-zol RNA extraction kit (Zymo Research, Irvine, CA, USA) according to manufacturer’s recommendations. cDNA was synthesized using 0.5 µg total RNA using RevertAid First Strand cDNA Synthesis kit (Thermo Fisher Scientific™, Milford, MA, USA). Real-time polymerase chain reaction (RT-PCR) was performed using TB Green Advantage qPCR Premix (Clontech, Mountain View, CA, USA) in MX 3000P detection system (Stratagene, AH diagnostics, Stockholm, Sweden). Expression of target genes relative to GAPDH were determined using 2^−ΔΔCT^ method. Experimentally validated PCR primers from PrimerBank (https://pga.mgh.harvard.edu/primerbank/) (accessed date: 15 June 2021) were used for validation (Table 4).

### 4.9. Statistical Analysis

Statistical analysis of RNA-seq data was performed using DESeq2 in the statistical environment of R (version 4.0.2) and visualization was conducted using either Qlucore Omics Explorer (version 3.6 (33)) or R program. Box plots represent the median (25–75 percentile) and the whiskers extend from the minimum to the maximum levels. Dots represent sample expression values normalized to mean = 0, variance = 1 for RNAseq data or sample values for other experiments. Significant differences between two groups were evaluated using the student’s *t*-test. *p* < 0.05 was considered statistically significant and for RNAseq data p adjusted (padj) value indicates *p* value after correction using Bonferroni method. *n* represents number of animals or experiments per group.

## 5. Conclusions

Our results show that sepsis caused massive transcriptional changes and that these changes differ in capEC and PCV. We believe that beyond the discussion addressed here, our data will provide extensive resources for discovery of additional genes and pathways involved in other inflammatory lung diseases. In conclusion, through transcriptomic analysis of lung capEC and PCV, we have identified several new genes and possible pathways involved in leukocyte recruitment, dysfunction of endothelium and disruption of homeostasis. We propose that targeting some of the identified pathways or transcription factors regulating the above-discussed functions could be beneficial to ameliorate sepsis-induced organ damage in experimental and clinical settings.

## Figures and Tables

**Table 1:** Top inflammation-related enriched KEGG pathways using top 500 DEGs from CapEC.

KEGG Pathway Term	Gene Count	*p*-Value	Fold Enrichment	Bonferroni	FDR
mmu04668: TNF signaling pathway	22	4.5 × 10^−12^	6.8	1.0 × 10^−9^	8.5 × 10^−10^
mmu04621: NOD-like receptor signaling pathway	10	3.7 × 10^−5^	5.9	8.4 × 10^−3^	1.7 × 10^−3^
mmu04620: Toll-like receptor signaling pathway	13	3.8 × 10^−5^	4.3	8.5 × 10^−3^	1.7 × 10^−3^
mmu04210: Apoptosis	10	5.7 × 10^−5^	5.6	0.012	2.1 × 10^−3^
mmu04064: NF-kappa B signaling pathway	12	1.2 × 10^−4^	4.1	0.027	3.3 × 10^−3^
mmu04060: Cytokine-cytokine receptor interaction	18	7.8 × 10^−4^	2.5	0.161	0.011
mmu04010: MAPK signaling pathway	17	3.0 × 10^−3^	2.2	0.49	0.028
mmu04115: p53 signaling pathway	8	3.4 × 10^−3^	4.0	0.53	0.028
mmu04350: TGF-beta signaling pathway	9	3.4 × 10^−3^	3.5	0.54	0.028
mmu04062: Chemokine signaling pathway	14	5.2 × 10^−3^	2.4	0.68	0.037

**Table 2:** Top inflammation-related enriched KEGG pathways using top 500 DEGs from PCV.

KEGG Pathway Term	Gene Count	*p*-Value	Fold Enrichment	Bonferroni	FDR
mmu04668: TNF signaling pathway	20	1.1 × 10^−10^	6.5	2.5 × 10^−8^	2.27 × 10^−8^
mmu04064: NF-kappa B signaling pathway	14	2.5 × 10^−6^	5.1	5.6 × 10^−4^	1.2 × 10^−4^
mmu04620: Toll-like receptor signaling pathway	14	4.1 × 10^−6^	4.9	8.8 × 10^−4^	1.5 × 10^−4^
mmu04621: NOD-like receptor signaling pathway	10	2.4 × 10^−5^	6.2	5.3 × 10^−3^	6.7 × 10^−4^
mmu04060: Cytokine-cytokine receptor interaction	20	4.3 × 10^−5^	2.9	9.3 × 10^−3^	1.0 × 10^−3^
mmu04210: Apoptosis	8	1.3 × 10^−3^	4.7	0.249	0.016
mmu04514: Cell adhesion molecules (CAMs)	13	1.8 × 10^−3^	2.8	0.335	0.022
mmu04630: JAK-STAT signaling pathway	11	7.3 × 10^−3^	2.7	0.794	0.065
mmu04066: HIF-1 signaling pathway	9	7.5 × 10^−3^	3.1	0.805	0.065
mmu04350: TGF-beta signaling pathway	8	9.4 × 10^−3^	3.3	0.871	0.072

**Table 3:** Top 5 transcriptional factor target (TFT) gene sets in lung capEC and PCV.

GSEA Name of TFT	DEGs (*p* < 0.05)
capEC	
GSEA_CREL_01	*BCL3, BIRC3, CASKIN1, CDC42SE1, CLDN5, CLOCK, COL11A2, CTDSP1, CYP2D6, FUT7, GPBP1, IER3, IER5, ITPKC, MAP3K8, MIDEAS, MOB3C, NFKBIA, PIK3R3, RAP2C, RBMS1, REL, RELB, S100A10, SDC4, STAT6, TACC1, TCF7L2, UBE2D3, UPF2, YWHAQ, YWHAZ, ZFAND3, ZNF593*
GSEA_NFKAPPAB65_01	*BCL3, BIRC3, CDC42SE1, CLDN5, CLOCK, COL11A2, CTDSP1, CYP2D6, FGF17, FUT7, GPBP1, HSP90B1, IER3, IER5, ILK, ITPKC, MAP3K8, MIDEAS, MOB3C, NFKBIA, NFKBIB, RAP2C, RBMS1, REL, RELB, RND1, SDC4, STAT6, UBE2D3, UPF2, YWHAQ, YWHAZ*
GSEA_ZNF791_TARGET_GENES	*ADA, BRWD1, CWC25, CYP2D6, FES, GIT2, RND1, SDC4, STAT6, ZNF827*
GSEA_TTGCWCAAY_CEBPB_02	*BNC2, CEBPB, HOXA5, KLF5, MIDEAS, NFKBIE, NRP2, RHOB, SLC7A11, TRIB1, VCAN*
MIR649	*ACER3, BICRAL, CACNA1H, CCDC6, GAPVD1, INPP5K, KPNA1, MEX3C, MOB1B, NUFIP2, RFX7, SENP5, STT3A, ZNF281*
PCV	
GSEA_GATA6_01	*ACKR1, BCL6, CTCF, ECHDC2, ERRFI1, FBRS, FOSL2, GPBP1, IGFBP5, KIZ, LHX6, LIMS1, LIX1, MID1, NOCT, NRAS, PCDH9, PFKFB1, PLAGL2, PSD4, SALL2, SLC39A14, SMAD5, TACC1, TGIF1, TGIF2, TPH2, UBE2H, WAPL, XPO6*
GSEA_TTGCWCAAY_CEBPB_02	*CSDE1, DBH, GSR, IDH1, IP6K2, MIDEAS, NFKBIE, RHOB, TRIB1, VCAN*
GSEA_MIR4769_3P	*BCL6, CCDC6, ERBIN, NFKBIE, PAK2, PTEN, WAPL*
GSEA_MIR362_5P	*ATF1, CPEB4, EDEM1, IDH1, LARP1B, PEG10, PLAGL2, RBM27, RPS6KB1, SNX18, SYNC, ADAM9, ATF1*
GSEA_CCTGAGT_MIR510	*CAPZA2, IP6K2, WAPL, ZHX2*

**Table 4:** List of primers for validation by qRT-PCR.

Mouse Gene Name		Primer Sequence (5′ -> 3′)	Length	Tm	Location
*Icam1*	Forward Primer	GTGATGCTCAGGTATCCATCCA	22	61.3	74–95
	Reverse Primer	CACAGTTCTCAAAGCACAGCG	21	62.0	286–266
*Mid1*	Forward Primer	CTGTGACGGCACCTGTCTC	19	62.3	1293–1311
	Reverse Primer	AAACGGCTGACTGTTGGTCTT	21	62.2	1497–1477
*Pim1*	Forward Primer	CTGGAGTCGCAGTACCAGG	19	61.4	100–118
	Reverse Primer	CAGTTCTCCCCAATCGGAAATC	22	60.7	240–219
*Enc1*	Forward Primer	CTGTTTCATAAGTCCTCCTACGC	23	60.4	64–86
	Reverse Primer	CACCACTGAACATGGCTTCG	20	61.3	232–213
*Il6*	Forward Primer	CCAAGAGGTGAGTGCTTCCC	20	62.2	462–481
	Reverse Primer	CTGTTGTTCAGACTCTCTCCCT	22	60.8	579–558
*Selp*	Forward Primer	CATCTGGTTCAGTGCTTTGATCT	23	60.5	69–91
	Reverse Primer	ACCCGTGAGTTATTCCATGAGT	22	60.8	173–152

## Data Availability

RNAseq data are deposited at Gene Expression Omnibus (GEO) database of NCBI (National Center for Biotechnology Information). (GEO Accession: GSE169532).

## Supplementary Materials

The following are available online at https://www.mdpi.com/article/10.3390/ijms221910181/s1.
