# Supplementary material for: Transcriptomic Analysis Reveals Differential Expression of Genes between Lung Capillary and Post Capillary Venules in Abdominal Sepsis
Source: Int J Mol Sci. 2021 Sep 22;22(19):10181. doi: 10.3390/ijms221910181 (PMC8507973; doi:10.3390/ijms221910181)

Transcriptomic analysis reveals differential expression of genes between lung capillary and post capillary venules in abdominal sepsis

Milladur Rahman#, Zhiyi Ding#, Carl-Fredrik Rönnow, Henrik Thorlacius*

Department of Clinical Sciences, Malmö, Section for Surgery, Lund University, 20502 Malmö, Sweden

---

Supplementary Figure S1. FACS plot showing gating strategy of lung blood endothelial subsets. Forward side scatter weight versus forward side scatter height, sideward scatter weight versus sideward scatter height were used to exclude doublets, live/dead staining and a pool lineage markers (CD11a, TER-119, CD45) versus forward side scatter was used to exclude dead and lineage positive cells. Blood EC (BECs, CD31+ cells) were separated from lymphatic EC (CD31+gp38+) and fibroblastic reticular cells (CD31-gp38+) by plotting CD31 versus gp38. Finally, blood capEC (CD31+Icam1+Vcam1-) and PCV (CD31+Icam+Vcam+) were sorted from sham and CLP animals for RNA extraction. Purity of sorted cells were confirmed over 97%.

Supplementary Figure S2. (A) Principal component analysis showing transcriptional diversity in resting (sham) capEC and PCV. (B) Top 500 variables in PCA plot ($p \leq 6.1e\text{-}06$, $padj \leq 1.9e\text{-}04$). Percentage in the parenthesis shows total variability calculated for each principle component (1, 2, 3). (C) Hierarchical clustering of top 100 differentially expressed genes plot ($p \leq 1.9e\text{-}15$, $padj \leq 2.9e\text{-}13$) in the lung sham capEC and sham PCV based on log_2 normalized expression values. P value indicates statistical significance and padj value indicates p value after correction using Bonferroni method.

Supplementary Figure S3. (A,B) Principal component analysis showing samples and variables (top 500) of capEC after induction of sepsis, ($p \leq 0.001$, $padj \leq 0.04$). (C,D) Principal component analysis showing samples and variables of PCV after induction of

sepsis, ($p \leq 0.001$, padj ≤ 0.05). (E) Principal component analysis showing all samples (4 groups) containing 500 top variables, p=0.002, padj=0.06. Variables are colored based on fold change. Percentage in the parenthesis shows total variability calculated for each principle component (1, 2, 3). P value indicates statistical significance and padj value indicates p value after correction using Bonferroni method.

Supplementary Figure S4. Top signaling pathway from KEGG analysis using top 500 DEGs ($p \leq 0.001$, padj ≤ 0.04) of capEC. Gene with significant differential expression in the lung capEC are marked by red asterisks in the top TNF signaling pathway. For KEGG pathway analysis mouse genes are converted to human orthologs as described in the material and method section. Pathway chart was created using online tool DAVID.

Supplementary Figure S5. Top signaling pathway from KEGG analysis using top 500 DEGs ($p \leq 0.001$, padj ≤ 0.05) of PCV. Gene with significant differential expression in the lung PCV are marked by red asterisks in the top NF-kB signaling pathway. For KEGG pathway analysis mouse genes are converted to human orthologs as described in the material and method section. Pathway chart was created using online tool DAVID.

Supplementary Figure S1

Pre-sort

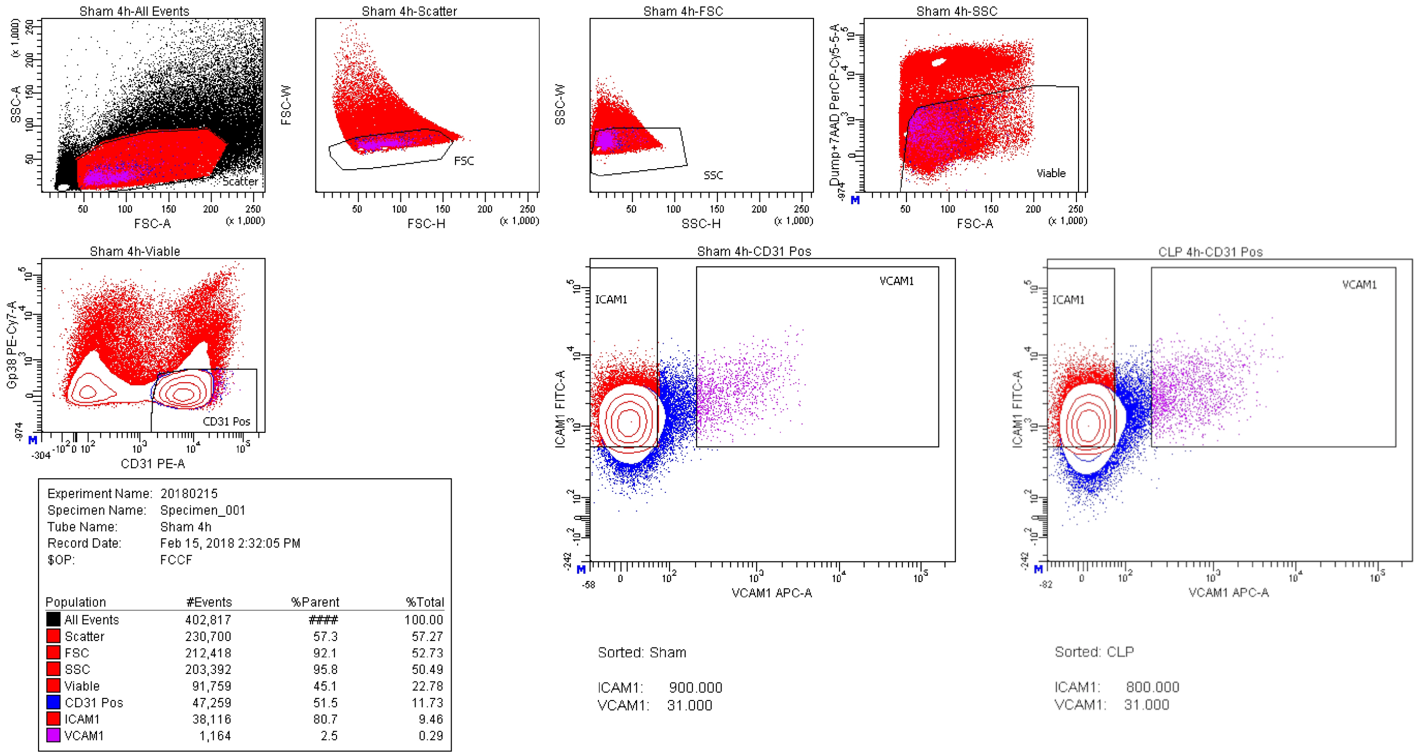

Experiment Name: 20180215
Specimen Name: Specimen_001
Tube Name: Sham 4h
Record Date: Feb 15, 2018 2:32:05 PM
$OP: FCCF

Population	#Events	%Parent	%Total
All Events	402,817	####	100.00
Scatter	230,700	57.3	57.27
FSC	212,418	92.1	52.73
SSC	203,392	95.8	50.49
Viable	91,759	45.1	22.78
CD31 Pos	47,259	51.5	11.73
ICAM1	38,116	80.7	9.46
VCAM1	1,164	2.5	0.29

Sorted: Sham

ICAM1: 900.000
VCAM1: 31.000

Sorted: CLP

ICAM1: 800.000
VCAM1: 31.000

Post-sort capEC

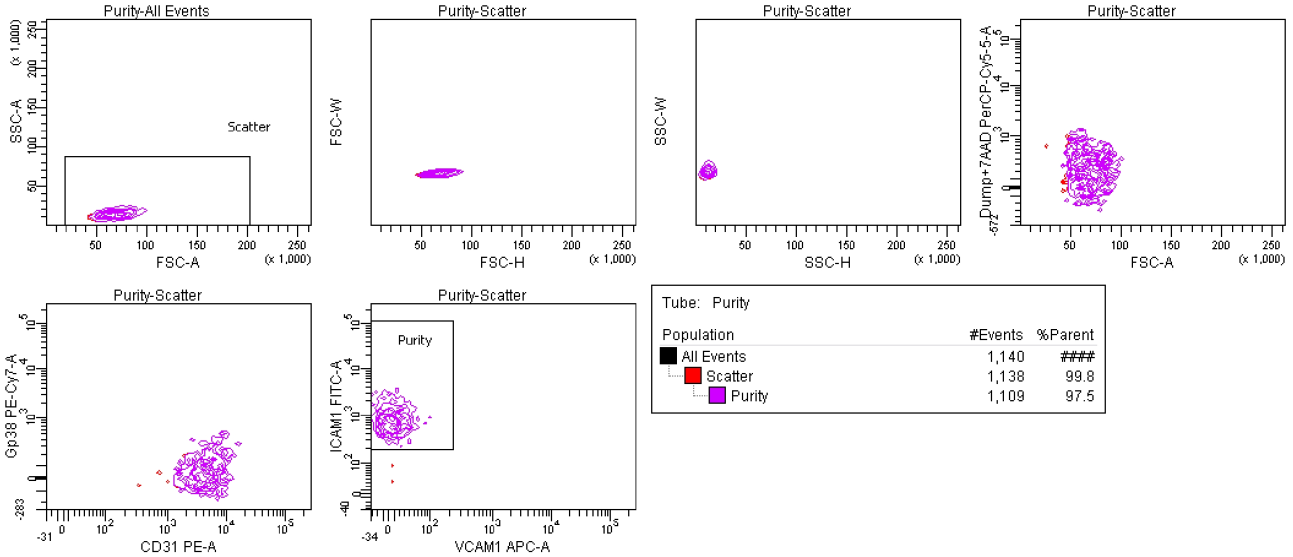

Tube: Purity

Population	#Events	%Parent
All Events	1,140	####
Scatter	1,138	99.8
Purity	1,109	97.5

Supplementary Figure S2

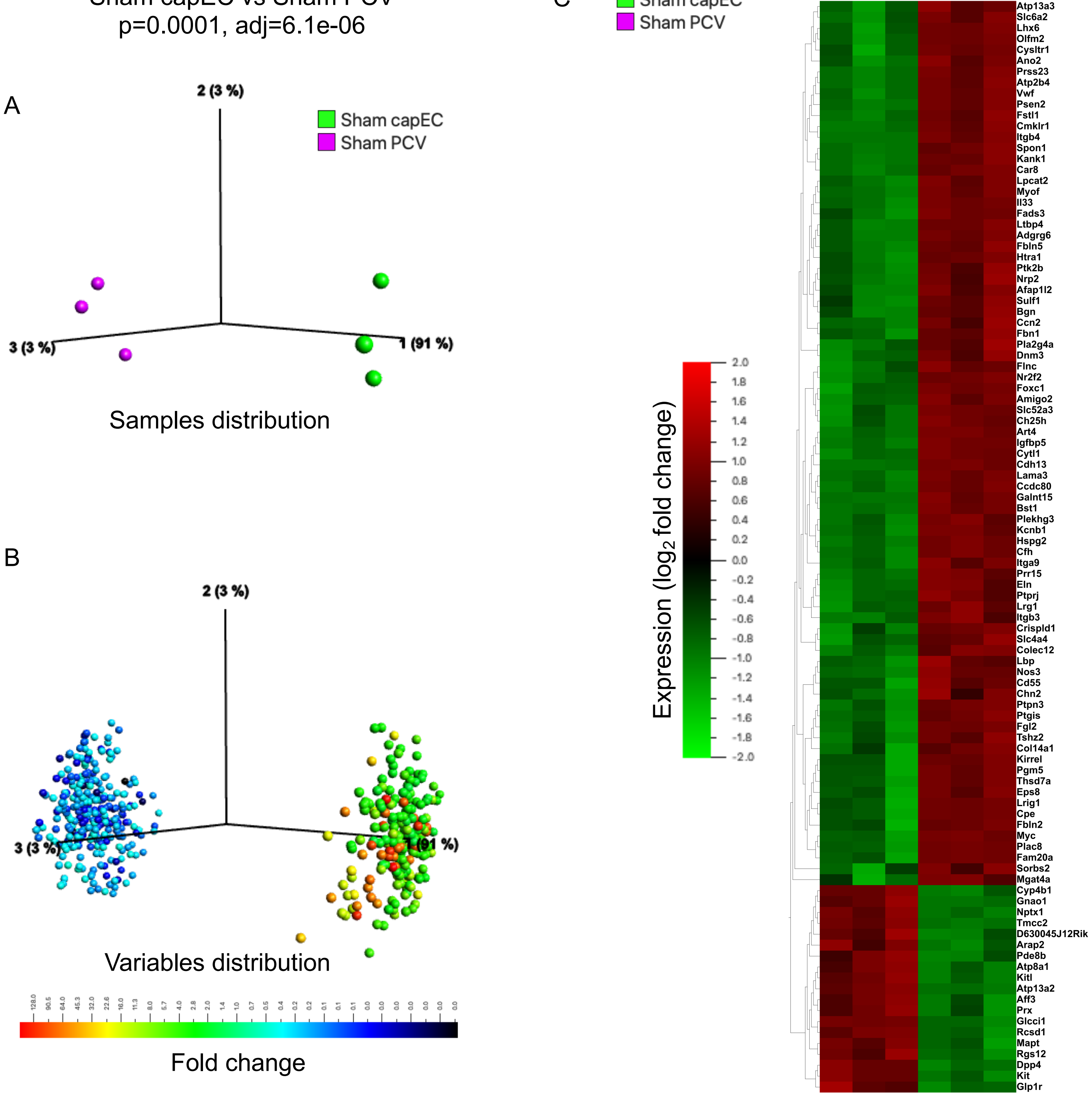

Supplementary Figure S3

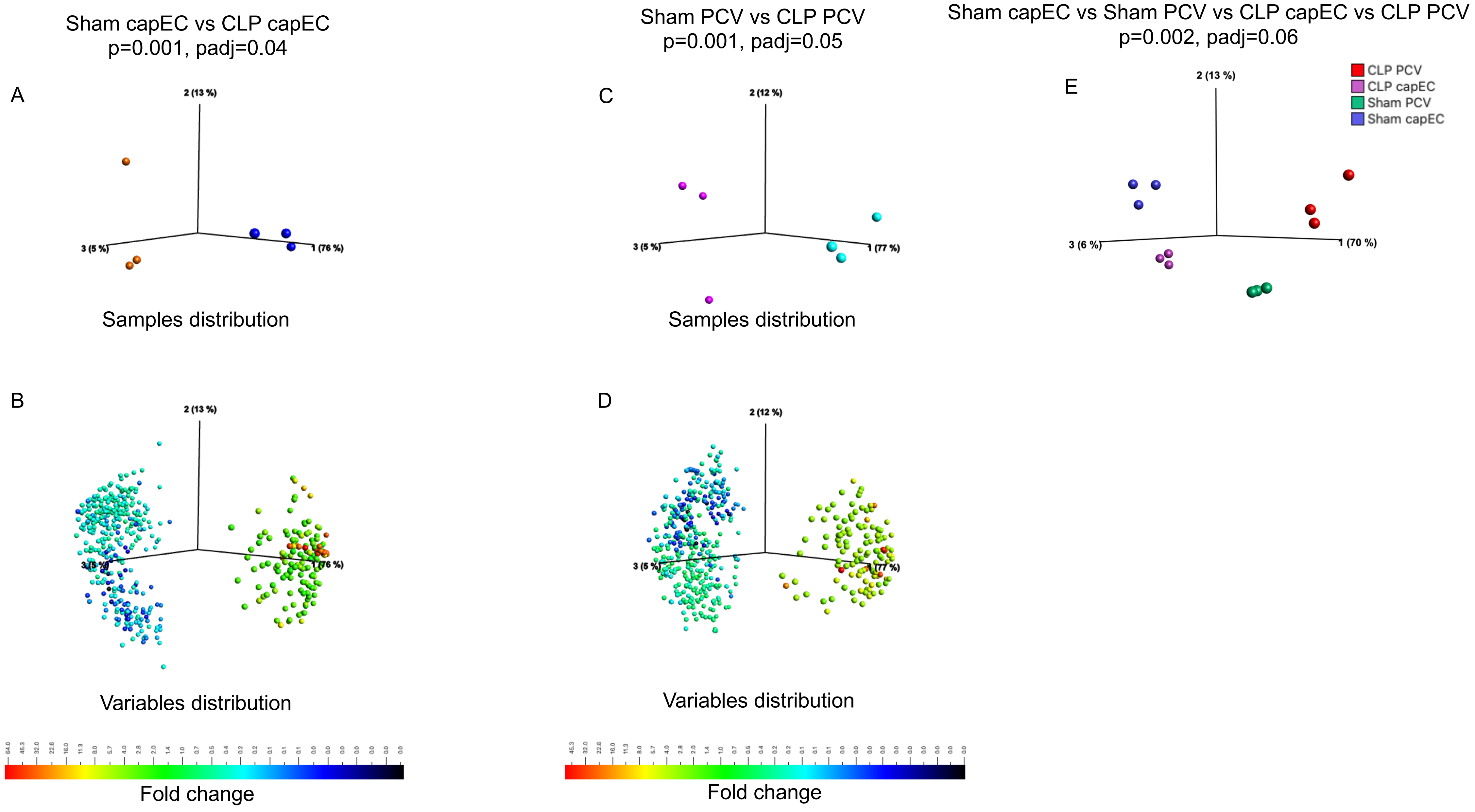

Supplementary Figure S4

Top enriched pathway using top 500 DEGs of capEC

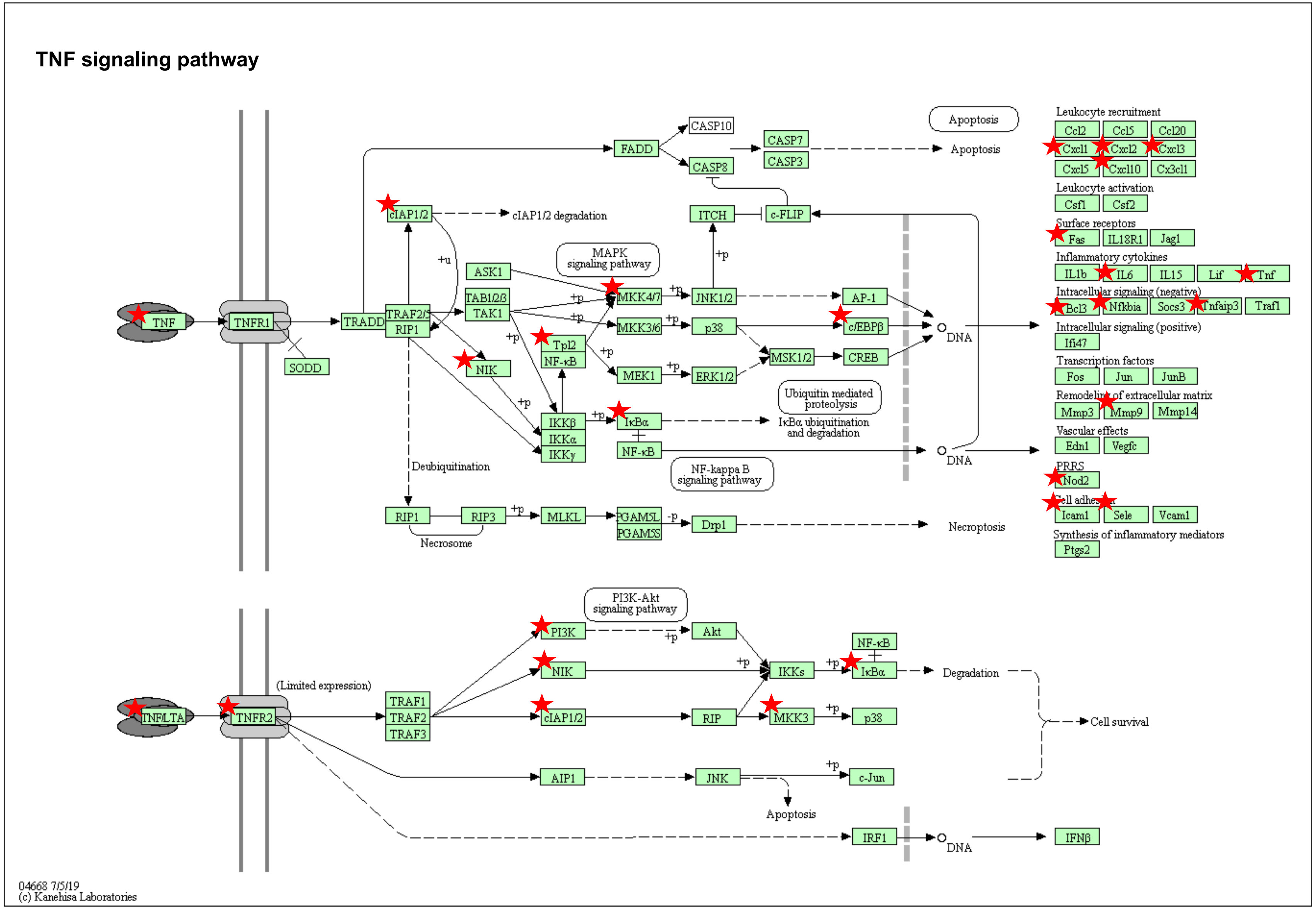

Supplementary Figure S5

Top enriched pathway using top 500 DEGs of PCV

NF-kappa B signaling pathway

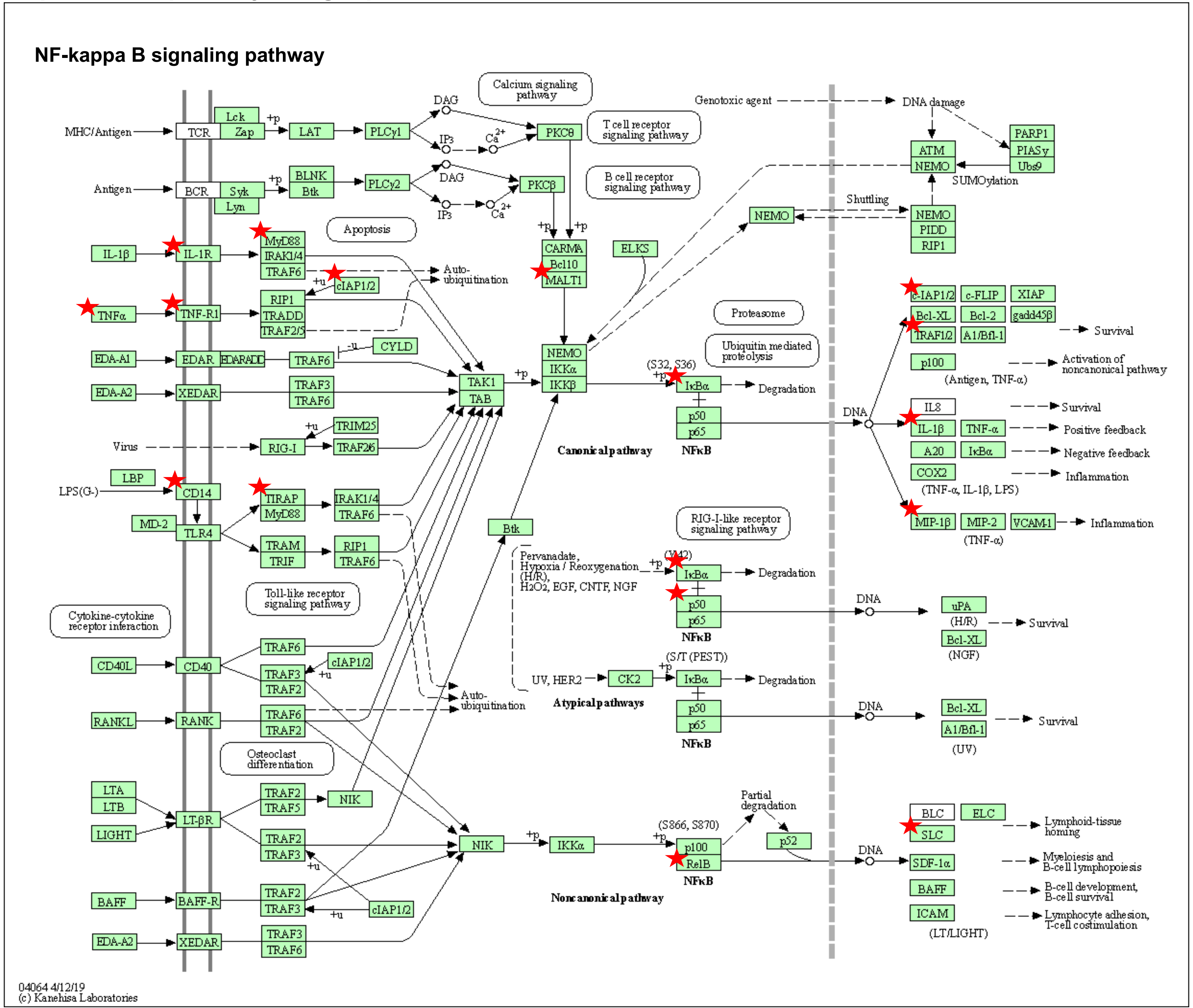

Supplement: Supplementary file 1 [file ijms-22-10181-s001.zip › Supple legends and figs.pdf]
